# “A Fire in My Belly”: The Health of Community Workers Who Support Women Survivors of Intimate Partner Violence

**DOI:** 10.1177/10778012251319317

**Published:** 2025-02-09

**Authors:** Tara Lundy, Joanne Crawford

**Affiliations:** 1 Department of Nursing, 7497 Brock University, St. Catharines, Ontario, Canada

**Keywords:** intimate partner violence, community worker, health, wellness, qualitative description

## Abstract

Nonprofit community-based workers (CWs) provide vital support to women survivors of intimate partner violence (IPV), and they are repeatedly exposed to secondary trauma in their everyday work lives which may influence health and wellness. Guided by the salutogenesis model, this qualitative study explored 19 CWs’ work experiences in relation to health and wellness in the Niagara Region, Canada. Five themes were generated using thematic analysis: (a) mental processing and alternations; (b) unmanageable structural challenges; (c) women empowering women; (d) unique ways of coping; and (e) recommendations for system improvements. Implications for intersectoral collaboration, health promotion, and chronic disease prevention are discussed to inform the development of tailored support strategies for CWs.

## Background

Intimate partner violence (IPV) is a worldwide public health problem that causes physical and psychological harm (World Health Organization [WHO], 2021) and disproportionally affects women who experience frequent and severe forms of IPV. The WHO (2021) estimates that nearly one-third of women between ages 15 and 49 have experienced some form of physical or sexual IPV. In 2018, 44% of Canadian women reported some form of IPV since the age of 15 and were about 4 times more likely to be killed by an intimate partner than males (Canadian Femicide Observatory for Justice and Accountability, 2020; Statistics Canada, 2021). Specifically, in Ontario, 24.4% of women reported physical and sexual assault committed by intimate partners in 2018; however, this did not consider the emotional violence committed by intimate partners (Statistics Canada, 2021). Local community-based organizations bear the greatest responsibility for supporting IPV survivors in immediate and long-term transitions. Services are provided by community-based workers (CWs) employed in nonprofit settings who are often the first point of contact for women IPV survivors, and they provide extensive emotional, instrumental, informational, and appraisal support (Lundy & Crawford, 2024). Nonprofit settings are typically organizations that are not publicly funded or bound by government policy or practice. CWs may be employed in emergency shelters, crisis hotlines, victim advocacy and support services, women's services/aid, and outreach agencies (Jirek, 2020; Lundy & Crawford, 2024). The demand for CWs' services has dramatically increased in the last few years because IPV incidents have escalated. For example, in the Niagara Region, a mid-sized city of 477,941 in Ontario (Statistics Canada, 2022), local emergency shelters for women reported an alarming demand for services. From 2020 to 2021, emergency shelters received a combined total of over 12,421 crisis/support calls, reported nearly 300 shelter stays, and one shelter reported a 91% increase in demand across all areas of programming since the COVID-19 pandemic (Birchway Niagara, 2022; Gillian's Place, 2022). Given that working life conditions is a key social determinant of health (WHO, 2023) that intersects with other determinants, this calls attention to the indirect effects of CWs being exposed to traumatic acts of violence in their everyday work life, which may subsequently impact their health, wellness, and ability to cope (Lundy & Crawford, 2024; Rossiter et al., 2020).

### Diverse Support Worker Experiences of IPV-Related Work

To explore the health and wellness outcomes of IPV support workers, a narrative review was undertaken. A total of 34 international papers written in English and published from 2002 to 2023 were included; four themes generated reflect that IPV support workers: (a) were employed in diverse settings with unique roles, qualifications, and responsibilities; (b) experienced meaningful and altruistic benefits; (c) reported adverse health outcomes; and (d) reported coping strategies such as workplace accommodation and self-care to be helpful (Lundy & Crawford, 2024). IPV support workers were employed in diverse settings, for instance, government-based systems, for-profit private services, or nonprofit community organizations. The education and credentials required also varied across the workplace settings as did the amount of time spent directly supporting survivors. A sense of purpose and fulfillment (Alani & Stroink, 2015; Maquibar et al., 2023), and compassion satisfaction (Kulkarni et al., 2013) were important benefits identified by IPV support workers. However, much of the literature reported experiences of adverse health outcomes such as psychological distress and physiological discomfort (AbiNader et al., 2020; Bell, 2003; Colombini et al., 2013; Garimella et al., 2002; Laisser et al., 2009; Maquibar et al., 2023; Van der Wath et al., 2013), interpersonal social challenges (Bell, 2003; Goldblatt et al., 2009), burnout (Alani & Stroink, 2015; Bemiller & Williams, 2011; Kulkarni et al., 2013; Watson et al., 2017; Wilson et al., 2018, 2021; Wood et al., 2019), as well as secondary traumatic stress and compassion fatigue (Bell, 2003; Ben-Porat, 2015; Brown et al., 2020; Voth Schrag et al., 2022). Several studies reported experiences of low job satisfaction, occupational stress, and high turnover intentions in the IPV field (Voth Schrag et al., 2022; Wood et al., 2019, 2020). Engaging in self-care and leisure-based activities (Alani & Stroink, 2015; Kulkarni et al., 2013; Van der Wath et al., 2016; Wachter et al., 2020), having available supports (Brend & MacIntosh, 2021; Choi, 2011), and workplace autonomy (Brend & MacIntosh, 2021) were found to be effective coping strategies in combating IPV work stressors. Evidence also suggests that IPV CWs may connect more deeply with survivors’ trauma as they develop long-term relationships to support survivors’ needs with ongoing challenges (Lundy & Crawford, 2024; Rossiter et al., 2020). Ellis and Knight (2018) suggest that IPV service workers may use empathetic engagement as a tactic to connect with survivors’ feelings and experiences in order to show understanding, compassion, and support them more effectively. However, empathetic engagement may be harmful if workers experience difficulty in distinguishing their own feelings from others and separating their professional and private lives, thereby disrupting their ability to control emotional responses (Ellis & Knight, 2018). Hochschild's (2012) concept of emotional labor may also explain this phenomenon, which partly involves the ability to manage or suppress distressful emotions to create a public display while preforming a job role and to produce a desired emotional state of mind from service users. As emotional laborers, IPV CWs are responsible to process and discuss survivors’ distressing experiences, and interact with them to gain trust, create a sense of safety, and show support, empathy, and civility. Further, nonprofit IPV CWs may not have adequate access to supportive resources and education to assist in their health and wellness, which may contribute to challenges in coping with IPV work stressors (Lundy & Crawford, 2024). Unfortunately, many authors in the review did not clarify the workplace context, which makes it challenging to determine individual or structural factors that influence work stressors and health outcomes because of the paucity of literature. Therefore, the current study used qualitative research design to gain an in-depth understanding of nonprofit CWs’ work experiences of supporting women IPV survivors, including coping strategies and recommendations that may be used to enhance health and wellness of this population in Ontario, Canada.

Antonovsky's Salutogenesis Theoretical Model (STM, 1987) adapted by Jenny et al. (2022) in the context of work was chosen for this study because it aligns with the concepts of health, wellness, and coping. STM represents a holistic approach used in public health promotion and wellness to understand the processes of managing life-stress experiences and strategies that may be needed to improve health (Antonovsky, 1987). In this model, health is relative on a continuum between two poles of complete wellness and illness as perceived by the individual. Jenny et al.'s (2022) model aligns with the research question in that it enables one to explore what factors, resources, or processes (generalize resistance resources) help individuals—in this study, individuals employed in nonprofit organizations—to cope with work stressors (generalized resource deficits). A workers’ sense of coherence (SOC) influences how they perceive their work to be meaningful, manageable, and comprehensible. It is the balance between work stressors, coping processes, and a worker’s SOC that influences their wellness and shapes their position on the health continuum. The STM (Antonovsky, 1987) has been widely used in other research studies for health promotion among workers. For example, the model was used to explore burnout, work engagement, and wellness among trauma counselors in Fourie et al.'s study (2008), job satisfaction among hospital healthcare workers (Rothmann, 2001), and stress symptoms among employees with diverse professional backgrounds (Albertsen et al., 2001). The adapted model was the guiding framework used for interview question formulation, and to construct the final thematic map of central themes and subthemes.

## Method and Procedures

A qualitative descriptive design was useful to gather rich data using low-inference interpretation to explore CWs’ experiences in relation to health and wellness (Neergaard et al., 2009). The study followed a naturalistic approach and was framed within an interpretivist view.

### Setting and Sample

Purposive sampling was used to recruit CWs who met the inclusion criteria: CWs who were employed in a Niagara nonprofit setting and worked directly with women survivors of IPV. CWs who only supported child survivors of violence were excluded because their experiences differed. Four organizations that met the criteria were approached by the primary author who had previously established relationships with the sites. Although the four organizations were nonprofit, they often derived revenue from government subsidies, grants, fundraising events, and community donations. A letter that outlined information on the study was sent to a contact from each organization. Invitation letters were distributed to CWs with the organization's permission and the primary author provided a presentation to CWs on the study.

### Data Collection and Analysis

A semistructured interview guide with open-ended questions was used to give some control and freedom to CWs when sharing their experiences. The interview questions centered on: (a) how CWs’ work experiences influence health and wellness; (b) how CWs perceive their work to be meaningful, manageable, and comprehensible; (c) helpful coping factors and processes; and (d) recommendations to enhance health and wellness. All interviews were audio and/or videorecorded with consent. Participants who volunteered were asked to select their interview date, time, and location (in-person in a private office or virtual) that best suited their needs and comfort level. Data analysis was achieved simultaneously with data collection by following Braun and Clarke's (2012) thematic analysis. Data analysis was managed manually through Microsoft Word for transcribing data.

### Ethics and Rigor

Relational and procedural ethics were maintained by obtaining ethics clearance from Brock University Research Ethics Board. Identification codes were assigned to maintain confidentiality and CWs were required to sign a consent form and complete a sociodemographic questionnaire prior to the interview. Several strategies were employed to demonstrate data trustworthiness. To achieve credibility, member checking was implemented by summarizing key ideas addressed in the interviews for CWs immediately after the interview, and when the primary author presented the findings back to each of the four organizations (Ravitch & Carl, 2021). Peer debriefing to uncover biases and receive critical feedback was frequently implemented between authors. Diverse transcripts were independently reviewed, coded, and themes were assessed by the second author. To enhance transferability, a clear description of the sample, and context were provided. Analytical memos and a reflexive journal were maintained to reflect on researcher biases and positionality for data dependability and confirmability (Ravitch & Carl, 2021).

## Findings

**Figure 1.**
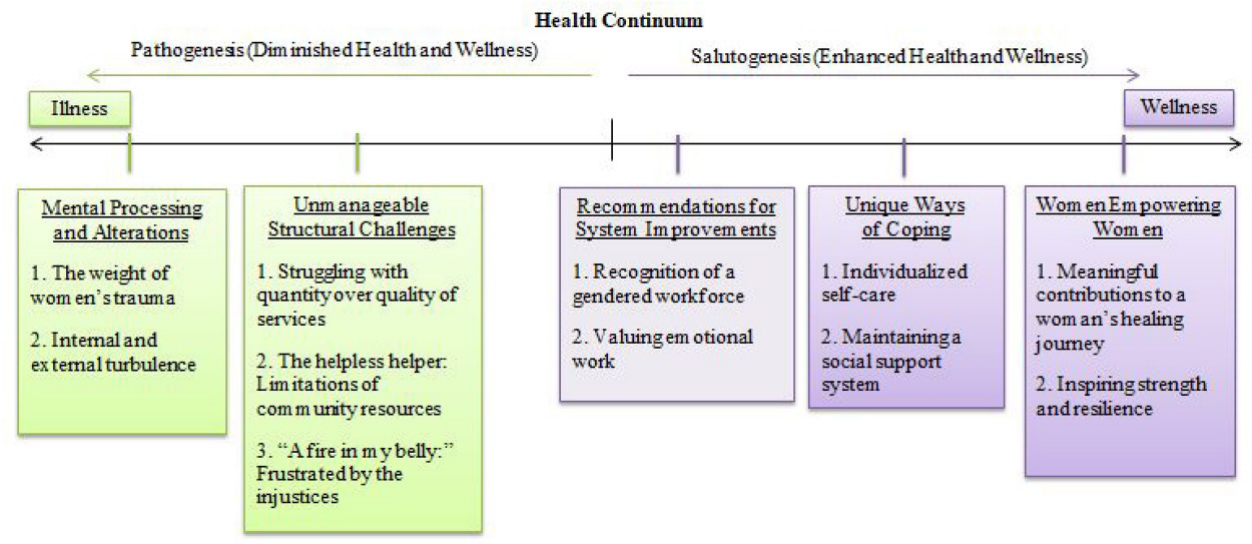
Five generated themes depicted on the salutogenesis health continuum.

A total of 19 CWs were interviewed. All CWs identified as female and they varied by age, relationship status, education, work experience, and position titles (see Table 1). Over one-third of CWs identified their position title as a counselor (36.8%); however, four CWs reported that they held multiple positions within their organization. Education varied as nearly half earned a university Bachelor's degree and almost all CWs (84.2%) had specific IPV training. Five interconnected themes and corresponding subthemes were generated and are depicted on a health continuum (Figure 1) following Antonovsky's (1987) STM. See Table 2 for a complete summary of themes, subthemes, and additional quotes.

**Table 1.** Participant Sociodemographics (*N* = 19) and Organization Services.

Sociodemographics	Percentage (%)
Age (in years)	
20-29	15.8
30–39	10.5
40–49	42.1
50–59	21.1
60+	10.5
Gender (female)	100
Relationship status	
Married/committed relationship	57.8
Separated/divorced	31.5
Single	10.5
Yearly income before taxes	
$30,000–$39,000	47.3
$40,000–$49,000	36.8
>$50,000	10.5
Declined to answer	5.2
Highest education obtained	
University bachelor's degree	47.3
College diploma	31.5
Postgraduate degree	21
Training specific to IPV	84.2
Employment role	
Counselor	36.8
Transitional housing support worker	21
Legal support worker	10.5
Antihuman trafficking support worker	10.5
Victim crises worker	5.2
Social worker	5.2
Parenting support worker	5.2
Executive director	5.2
	*M* (*SD*)
IPV-related work experience (in months)	127 (138)
Number of survivors supported (per week)	16.1 (7)
Number of hours directly supporting survivors (per week)	27.8 (8)

IPV = intimate partner violence.

**Table 2.** Themes, Subthemes (Italicized) and Associated Supporting Quotes Identified Through Thematic Analysis.

Theme or subtheme	Supporting transcript quotations
Theme 1: Mental processing and alterations	
*The weight of women's trauma*	“There's still things in my head that women have told me and shared with me that I’ll never forget. This one woman, her husband used to hold her over the stove, turned the stove on, and held her over until her skin would start to burn. Physically, even now it, makes me feel sick.” (CW16)
*Internal and external turbulence*	“I find it natural that I seem to sometimes become the person people always go to. I’m like, I’ve already done that at work, I can’t, I’m exhausted. I’m giving all my time, energy, and emotional mental capacity to strangers in a sense. Now I don’t have anything left to give to the people that I probably should be because they’re my family and friends. Even sometimes with the kids, after a long day I’m like, I just don’t want to talk, I just want to relax.” (CW03)
Theme 2: Unmanageable structural challenges	
*Struggling with quantity over quality of services*	“I do find it stress-inducing in the sense of again, that juggle between quality and quantity because it's not okay to just give mediocre service to hundreds, versus quality service to let's say 60 (women survivors).” (CW12)
*The helpless helper: limitations of community resources*	“With housing, sometimes it can take years … (women) can be homeless for 10 years or something waiting for a place … so I think helplessness in regards to situations like that where you want to support someone, you know they’re asking for help to get housing, but it's kind of out of your hands as well.” (CW05)
*“A fire in my belly”: frustrated by the injustices*	“Criminal harassment pieces are very challenging … it seems like there's just so little that the law can do and (the women) are just having their partners getting arrested again and again then getting release the next day. So sometimes safety planning can be frustrating in that regard because you’re asking this poor client to do all these things and make all these changes, when it's in no way their fault that they’re in that position. I feel frustrated going through this with women so I can only imagine how frustrated they feel having to make all these changes.” (CW09)
Theme 3: Women empowering women	
*Meaningful contributions to a woman's healing journey*	“[Supporting survivors] makes me feel like I’m actually there during the helpful moment in their long-term journey of healing…giving them resources and knowing that I’ve gotten them the counseling that they may need to start the process of healing can feel really good.” (CW07)
*Inspiring strength and resilience*	“(Supporting survivors) can make me realize that the things I do in my personal life have helped me get to a good place of self-esteem or confidence, or I try not to take other people's negative words and things like that too harshly. So, hearing it makes me realize how far I’ve come in my own struggles … you know being put down I’ve had negative relationships myself, just to see where I’ve grown from. So, there's a positive side to it, making me recognize the growth in myself.” (CW02)
Theme 4: Unique ways of coping	
*Individualized self-care*	“I’m not much of a gardener, but I will go outside and work at it. Sometimes, you can’t control anything expect that little square of ground. There's processing in that and there's nothing more grounding than gardening. It's very sensory … it's a lesson, too. I can’t change the world, but I can introduce ideas just like I can introduce things to my garden and maybe they’ll work out.” (CW01)
*Maintaining a social support system*	“One of the biggest things is knowing that we can talk to our employer, I think that's super important. I’ve been in roles where I didn’t have that luxury. I think in this field, we definitely need to feel more comfortable to talk to our coworkers and supervisor with our own personal issues because like I said, we need to be mentally OK to do this role.” (CW02)
Theme 5: Recommendations for system improvements	
*Recognition of a gendered workforce*	“(Workplace independence) makes me feel good because I don’t feel that stress or that somebody's telling me that I have to do something. If I have a cancelation, I have independence or freedom to just work on the case notes, or other priorities that need to be done too, like checking in with my own emotional health.” (CW13)
*Valuing emotional work*	“It feels like lip service when those in power say how mental health matters and they’re putting this money into that … we are fully funded through the government yet there have been zero additional funds for us when it comes to our pay. And we do important work.” (CW11)

### Mental Processing and Alterations

The first theme captures experiences related to CWs’ struggles from processing the consequences of women's trauma, and how their work was internalized, which interfered with their way of being. This ultimately shaped their positioning toward the illness pole of the health continuum.

#### The Weight of Women's Trauma

All CWs shared how processing women's traumatic stories and seeing the horrific consequences of physical abuse manifested into unpleasant emotional and physical symptoms. Most CWs described feeling depressed, disheartened, or emotionally drained from hearing demoralizing stories of women's abuse daily. CW09 shared how the abusive stories she heard and the suffering of women affected her psychologically; she explained how these stories invoked such strong feelings of empathy that she felt “the weight of what (a woman's) going through” and reported feeling “emotionally drained at the end of the day.” Hearing the graphic details of the physical harm inflicted on women were described as “gut-wrenching” (CW12) and CWs expressed physical sensations such as feeling nauseated. Like symptoms of vicarious trauma, some CWs described experiencing panic attacks, sweating, anxiety, depression, or sleeping issues from seeing the aftermath of a woman's physical abuse, such as the bruises, burns, cuts, and strangulation injuries that were caused by a partner. For CW04, seeing these women troubled her to the extent that she needed medications to aid in her coping, such as antidepressants and sleeping medication, and she had to take an extended leave from work.

Several CWs struggled to emotionally comprehend how a human can inflict such physical violence on another; in relation to the STM (Antonovsky, 1987), these experiences eroded CWs’ ability to cognitively appraise their work circumstances as comprehensible. They revealed feeling disturbed and saddened as the physical abuse directed at women was becoming increasingly violent over time. CW01 fearfully anticipated hearing that one of her clients would end up dead, while CW06 shared disturbing experiences of various tools used by abusers to physically assault women. Moreover, crisis calls with women created stress and catastrophic thoughts, which fueled anxiety-related symptoms among CWs, for example, feelings of shakiness, nervousness, sadness, and feeling general unease. Experiences of disappointment and feeling heartbroken were reported when CWs saw a woman “revictimized” (CW09) because they returned to their abuser or entered a new abusive relationship, and these feelings were related to their own self-perceived inadequacies of their support. For many CWs, they questioned if they were making any difference in their role after seeing the same clients return to the organization for support.

#### Internal and External Turbulence

Although quite a few CWs did express feeling more grateful for their healthy connections with loved ones, nearly all the CWs described ways in which their work created apprehension and uncertainty, which interfered with their personal lives. Several CWs had trouble developing friendships or relating to others who do not work in the IPV field or understand the complex cycle of abuse. For instance, CWs reported feeling angry toward others who carried judgments about experiencing abuse. Because of her lens in regard to the reality of IPV, CW06 described feeling a “why bother attitude” during these judgmental conversations about IPV with friends, and felt that advocating for women in these situations was futile so she disengaged from these discussions. Some CWs struggled with bringing challenging work experiences home, which in turn, affected their mood and projected into unpleasant behaviors toward loved ones. CW02 explained that some days she had a harder time disconnecting from work at home, and that sometimes she “lashes out,” is overly quiet, or “snobby” toward her intimate partner because she is aggravated and frustrated by work-related issues. Likewise, CW14 described having difficult discussions with her husband because he felt she was directing her anger at him from dealing with “so many kinds of bad men” who abuse women. Others explained how they no longer had the mental capacity to be a venting outlet for their family or friends because they were emotionally drained, “checked out” (CW12), or “listened out” (CW11) from supporting women in crisis all day.

Because CWs were more attuned to the abusive realities of the world, this led to excessive worrying, a heightened sense of safety, and hesitancy in trusting others. About half of the CWs disclosed how their work made them hyperaware of red flags in others which, for some CWs, affected their own intimate relationships. For instance, CW03 described herself as “tainted” and “still single” because she saw “everything as a red flag.” Other CWs developed anxiety related to the possibility of client situations happening to them; for example, CW11 felt “so much anxiety” and questioned her trust with her partner after supporting a woman who was blindsided by her partner's 5 years of cheating.

### Unmanageable Structural Challenges

The second theme encapsulates the structural challenges that were not within the CWs’ control; this influenced their inability to view their work-related challenges as manageable and positioned CWs away from the health and wellness continuum.

#### Struggling With Quantity Over Quality of Services

Developing “quality connections” (CW11) and meaningful engagement with women survivors was important among CWs to establish therapeutic relationships and keep women focused on their healing journeys. However, CWs felt that insufficient staffing levels and increased workload demands did not allow them the time or capacity to develop relationships, provide personalized and quality support, and meet the needs of IPV survivors. This resulted in CWs feeling high pressure, stress, and overwhelmed with the number of cases, which led to exhaustion and put them at risk for burn out and compassion fatigue. For example, CW09 shared her frustrations when “there's a really heavy workload and you feel like you’re not giving people the care that they deserve.” In contrast, CW03 explained that because she must support more clients than she has the time for, she “may kind of stretch (herself) out” by trying to give every client at least a little bit of support as a way to assist them all. Because of the excessive workload demands, several CWs reported that they rarely took work breaks because they felt a sense of duty and commitment to give women the time required to provide meaningful support. Others described their work as high pressured when trying to manage multiple, time-sensitive, and complex client situations with limited staffing capacity. For instance, CW08 stated that CWs “work in crisis and support people in crisis,” and that “the workload is just so much. It's too much sometimes and you’re just trying to get to it as much as you can, and you just know there's more looming.” The CWs’ inability to manage their stressful workload manifested into unpleasant physical symptoms, such as headaches, body tension, and high blood pressure. CW07 developed irritable bowel syndrome that may have been in part triggered by work-related stress. CW08 struggled with weight gain because she was exhausted from work and lost interest in exercising. Other CWs reported psychological symptoms such as mental exhaustion, burnout, and irritability. CW06 explained she tended to feel less productive by the end of a chaotic week: “I just mentally slow down. I physically slow down. My brain almost hurts sometimes … it just aches and I start to get a bit forgetful.” She continued to explain that the heavy workload did not allow her the time to address the intricate details of women's increasingly complex cases, which in turn contributed to her feeling stressed and fatigued.

#### The Helpless Helper: Limitations of Community Resources

Nearly all the CWs expressed helplessness, guilt, and experienced a loss of motivation to do their work when there were obstacles to support women in moving forward from their abusive situations because of the limited capacity of community resources to meet the needs of IPV survivors. Although this was a structural issue not within their control, many CWs felt helpless and struggled with how to create safety plans for women with limited resources. For example, CWs struggled to find safe shelter for women because emergency shelters were often at capacity or the waitlist for affordable transitional/permanent housing was long. If CWs cannot secure housing out of the region or if survivors cannot live with friends or family, they unfortunately are sometimes left homeless or they feel forced to return to their abuser for shelter. In these instances, several CWs were left feeling helpless and felt like a failure. CW18 shared: “It's not an easy thing, so my sadness comes when (a woman) chooses to go back to that abusive situation. I feel like I’m failing them.” Other CWs felt helpless because of systemic issues related to limited government funding to support women's needs, such as funding to purchase home safety items like a security camera or deadbolts, or to cover dental coverage for significant injuries because of abuse. Although the availability of funding support for resources was out of their control, CWs often took the brunt of women's anger after being the “bearer of bad news” (CW03) and were blamed for “revictimizing” (CW06) women because they could not assist them to obtain a needed resource.

Several CWs discussed how mental health and addictions were interconnected with IPV, and thus, posed barriers for women in that this inhibited them from leaving abusive situations. Because it is not within the scope of their role, some CWs expressed feeling helpless in supporting women with mental illness and addictions because they lacked knowledge and training, and there was limited access to psychiatric resources in the community. They wished for improved competency and training in mental health and addictions to better support their clients. Moreover, CWs’ job is further complicated when survivors cannot receive proper mental health and addictions support because they may become ineligible to access other necessary resources, such as emergency shelters, due to the organization's admission guidelines. CW09 expanded on the difficulty of trying to navigate these tough situations with women who were banned from shelters because of drug use and aggressive behavior:

it's very hard in those situations, if a woman can’t come to our shelter because she's banned, she likely can’t go into the other shelter. She's probably banned there. She might be banned from all the homeless shelters, or they’re full. It's very difficult when someone has nowhere to go and unfortunately mental health goes along with victims of severe trauma.

As a result of the systemic limitations of community resources in relation to housing, funding supports, and mental health and addiction services, CWs struggled with navigating unique issues of diverse types of survivors and felt like a helpless helper.

#### “A Fire in My Belly”: Frustrated by the injustices

Feelings of frustration, powerlessness, sadness, and anger were emotions described by some CWs due to the injustices and lack of empathy for women who had to go through the legal system. Several CWs discussed their frustrations with the court process and their difficulty in supporting women through litigation abuse, which is when an abuser uses the legal system to continue to exert power and control over women. For instance, CW10 described her challenges of supporting a woman through an 8-year court battle over custody of her children; the women's abuser would delay court dates and was willing to pay large sums of money to hire lawyers who fought to delay the proceedings. This in turn left CW10 feeling powerless and stuck in how to support the woman. CW10 described these injustices toward women as a “dark cloud looming over the work that we do” and that it felt like “a game of chess” trying to support women through litigation abuse. For other CWs, it was saddening to see a woman's abuser repeatedly get released on bail, even after multiple abusive offenses. CW07 described these situations as “saddening and downing,” while CW13 expressed her anger toward the court process and injustices within the system:

Seeing (women) go through the court system, just being torn down, the injustices, and to watch some women, it's really hard. A fire in my belly just gets really large with that. There's a lot of times where I’m like, this is just unfair, it doesn’t seem right. This woman deserves so much more than this.

In fact, because “the system is dysfunctional and broken in so many ways” (CW11), and because of how long and demoralizing the process is for survivors, several CWs discouraged women from going to court if they did not need to. Some of these situations also created challenges for CWs with respect to safety planning for women, for example, when CWs or women were not notified when the abuser was released from jail, or when police did not follow up on the abuser's charges.

### Women Empowering Women

The third theme involves the meaningful and inspiring aspects of women empowering other women, as all CWs interviewed identified as female and supported other women who were experiencing abuse inflicted by men. These work experiences contributed to greater overall health and wellness by providing CWs a sense of meaning and inspiration to enhance self-reflection and growth.

#### Meaningful Contributions to a Woman's Healing Journey

Supporting women survivors brought all female CWs a sense of meaning and purpose. Many CWs felt that their work was gratifying, fulfilling, rewarding, and sometimes, these feelings superseded their daily work challenges. They described how they enjoyed and valued building trust, rapport, and establishing quality connections with women. Since survivors were frequently socially isolated because of their abuse, CWs felt privileged and honored to be the person that survivors were able to safely confide in during some of their most vulnerable moments. CW11 shared:

What's meaningful to me is being able to be a person that (women) feel like they can turn to … it means so much to me that they think of reaching out to me when they’re having a hard time, and that there is an opportunity for them to feel safe and just show who they are and invite me into their vulnerable internal world. I think it's a big privilege and I feel really honored to do that for (women).

Women empowerment was commonly described by CWs as a meaningful aspect of their work. For example, providing other women with the education and tools to develop self-worth and self-esteem, and to regain a sense of control felt gratifying for many CWs. Some CWs shared how they felt fulfilled from having the ability to put a smile on a woman's face during some of their most difficult times. Others found their work rewarding by helping women progress through their healing journeys and making an invaluable impact on women's lives from their relationships and continued support. Additionally, CWs felt their work was rewarding by having the opportunity to assist women to set and achieve goals, help keep them safe, and to show up for them in a way that is supportive and nonjudgmental.

#### Inspiring Strength and Resilience

The empowerment that CWs provided to women survivors was reciprocated; almost half of CWs indicated how inspiring it was to see survivors’ strength, bravery, and courage to move forward from an abusive situation. CW12 elaborated:

I get to see women show up for themselves. I get to hear them talk about their strengths and they show up with courage even when they don’t want to or when it's difficult. I think it's inspiring in terms of seeing their vulnerability. So that piece, that inspiring piece stands out for me.

In spite of being directly and repeatedly exposed to the tragedies of women's abuse, CW09 described how women's resiliency, strength, and how they “reshape themselves after tragedy” can be “encouraging” and created a positive balance in her work. Likewise, the women's resiliency encouraged some CWs to reflect in their own struggles and motivated them to become a stronger person when faced with personal challenges, such as when they encountered issues in their own relationships.

### Unique Ways of Coping

Engaging in effective, unique coping methods assisted CWs in managing their work-related stressors. The following subthemes describe CWs’ personal ways of coping that helped them when supporting women survivors; namely, individualized self-care, and maintaining a social support system of understanding individuals.

#### Individualized Self-Care

The CWs described several different self-care strategies that assisted them in coping with stressful work experiences. The most reported self-care strategy was to practice compartmentalizing emotions, which involved mentally identifying and separating triggering work feelings and thoughts from one's personal life. This strategy helped some CWs’ mental wellness by identifying personal stress triggers and recognizing when they need to decompress so that work and personal life could feel manageable. However, compartmentalizing emotions effectively was a skill that took years of practice; CW16 claimed it had taken her nearly 30 years to successfully achieve. Similar self-care strategies included practicing mindfulness, meditation, and yoga. These strategies assisted CWs in calming the nervous system and developing a positive mental space for work. Seven CWs also sought psychotherapy and counseling to assist with their mental well-being by discussing and processing their triggering work-related emotions and experiences.

Other CWs reported engaging in leisure-based activities to promote self-care and wellness, such as reading, cooking, listening to podcasts or music, crafting, or spending time with friends and family. Many CWs reported that exercising and spending time in nature assisted with stress relief, their ability to reset, and making sense of the world. For example, CW01 elaborated on how gardening was a “sensory” activity that gave her a sense of control when she felt she could not “change the world” and when situations at work did not feel manageable. Interestingly, several CWs shared that they disconnected from social media as a method of self-care; for instance, CW02 explained how consumed she felt in her work as her social media algorithms were displaying domestic violence-related material on her personal phone. Likewise, several CWs reported that they avoided watching violent television shows during their personal time as they were too triggering or aligned closely to the context of their work. CW01 shared:

People will ask me, “what do you watch on Netflix? Are you watching this, are you watching that?” No, I don’t like to watch things where people kill other people, stalk other people, or cut other people up. I need to spend that time relaxing. I need light stuff that lifts that away.

The CWs preferred watching happy and light-heartened television programs such as comedy, reality, or sports shows to laugh and relax.

#### Maintaining a Social Support System

Many CWs shared how helpful it was to receive social support and debrief with coworkers to cope with work-related stressors. Most CWs describe their coworkers as genuine, nonjudgmental, and open individuals who understood the context of their work-related stressors. For instance, CW09 stated: “my coworkers are lovely and very supportive. We can chat about things and vent, that's always helpful. That always makes you feel a little bit more sane.” Some CWs were able to bond over similar work situations and use humor therapeutically to lighten the mood and relieve stress. The majority felt comfortable to reach out to their coworkers and employer at any time, which many believed was an important aspect of working in a trauma-related field and be “mentally okay to do this role” (CW02). This assisted CWs in navigating tough client situations and provided reassurance and validation that helped alleviate some of the emotional burden of challenging survivor situations. The CWs explained they would benefit from managers and coworkers in continuing to reach out, complete check-ins, and regularly ask how workers were doing.

### Recommendations for System Improvements

This final theme captures CWs’ recommendations for system improvements that would assist them in their work by enhancing awareness of their role, needs, and overall health.

#### Recognition of a Gendered Workforce

Since the majority of IPV survivors are women, it is not surprising that women are predominately engaged in these CW roles. Many CWs revealed that they also carried the role as primary caregiver for their families as some had young children and several were single mothers. The gendered nature of the IPV workforce is important to recognize as women's work-related stress may compound the stress involved in balancing other social identities, such as a wife, mother, or primary caregiver. For instance, CW06 wished for more compassion and for people to better understand the challenges of being a woman, a mother, and working in an emotionally demanding role:

I feel like there's a lack of understanding on what I have to balance every single day with kids and home life and being everything … being the organizer, the taxi driver, the coordinator. Other women often feel the same way, they’re burning the candle at both ends sometimes and it's really hard to get that balance and not have mom guilt.

In recognizing the gendered IPV workforce, most CWs emphasized the value in having their managers appreciate workers’ independence and to facilitate flexible work hours. Some CWs explained that the quality of their work was better when they did not have the pressure or stress of feeling micromanaged, and their time was used more effectively when they could flex their own work hours. Some CWs expressed that they appreciated a hybrid work model where they had the option to independently work from home if they did not need to complete in-person work tasks; this reduced overall stressors that are inherent to holding multiple social identities. Having workplace independence and flexibility contributed to an enjoyable working environment and CWs feeling respected, less stressed, improved productivity, and overall health and wellness.

#### Valuing Emotional Work

The emotional burden of carrying survivors’ traumatic stories for CWs was clear. While individual coping strategies play an important role in protecting against work-related stress, many CWs emphasized structural or system-level changes that need to be made to promote their health and wellness. Most importantly, CWs described feeling devalued, unappreciated, and that the emotional and physical consequences of their work were unrecognized. CW02 explained, “we deal with a lot, we go through a lot, and we are really helping other people, so I think there's a lack of acknowledgment for that.” Most CWs felt that the work they do was not truly understood or valued because of the low wages and little to no health benefits provided in their compensation packages. Even with having the same level or advanced education, some CWs felt that their wages were low compared to workers with similar roles who were employed in government institutions, such as “Ontario Works or Family and Children's Services” (CW18). Yet, the emotional burden may be similar or heavier in some circumstances. CWs explained that their organizations struggled to find people to hire because many CWs were leaving nonprofit sectors to work in private practices or government institutions for higher pay and a better work–life balance. Receiving inadequate wages contributed to CWs feeling their work is undervalued, the stress of keeping up with inflation, and planning for retirement with no savings. Others experienced burnout from working a second job or extra shifts at their organization so they could cover expenses, which was particularly challenging for women who were single mothers. Moreover, several workers struggled with receiving little to no health benefits, for example, insufficient dollar amount for professional counseling to cope with work-related stress. For CWs who were single mothers, covering essential health expenses for their families within a single-income household created an additional burden; CW11 shared: “we don’t have any dental coverage, which is pretty expensive to cover for a family.”

CWs also expressed the need for increased ministry government funding to assist in facilitating manageable workloads. Several CWs felt that their organization was financially compensating them the best they could, however, “from a government standpoint” (CW13), being dependent on government grants to operate is not sufficient. Nonprofit organizations faced challenges related insufficient funds for full-time, permanent staff to meet the increasing service demands and complex needs of IPV survivors; this ultimately impacted their workload and contributed to their stress. CW08 shared: “our stats are increasing, but the staff has not increased … there's been the same amount of staff the entire time I’ve been here.” Several CWs believed that their work, as well as the needs of survivors, were not prioritized among the Ontario Government and the lack of funding reflected a systemic gender equality issue. CW17 shared: “I think it's because (our organization) is run by women for women. If this were happening with men, it would be an epidemic and we’d be getting funding.” Acknowledging the emotional burden of CWs’ work by valuing and appreciating these unique roles while also improving wages and benefits may encourage staff retention in nonprofit organizations. Additional funding to sufficiently staff nonprofits may assist in facilitating manageable workloads, preventing CW stress and burnout, and facilitating awareness of the necessity of these roles to the recovery of IPV survivors.

## Discussion

Exploring the experiences of CWs provided valuable and relevant insights anchored at both ends of the health and illness STM continuum. The benefits of supporting women IPV survivors may be seen as contributing to health because it elicited a sense of purpose and fulfillment, which has been reported among IPV support workers from diverse settings in other studies (Alani & Stroink, 2015; Maquibar et al., 2023). Additionally, CWs in this study reported a sense of empowerment from the resiliency of survivors. Although, the cognitive energy and effort required by CWs to work in this emotionally demanding role were also contributors to self-perceived adverse health outcomes that if not addressed may lead to risk for chronic diseases, such as depression, emotional exhaustion, burnout, stress, sleep disturbances, or chronic anxiety (Grandey & Sayre, 2019; Jeung et al., 2018; Kim, 2019). Emotional labor has been linked to adverse health conditions such as depersonalization, heart disease, hypertension, cancer, or memory loss (Jeung et al., 2018). The findings build on the concept of emotional labor, which involves the ability to manage, induce, or suppress emotions in order to sustain a certain semblance required to perform a job role (Hochschild, 1983, 2012). For instance, Hochschild provides an example of a judge who does emotional labor; while a judge may be exposed to stressful or disturbing incidents of murder or child rape, they are required to face such distressful emotions and manage an outward countenance of impartiality to perform their role (Hochschild, 2012). Hochschild (2012) explains how some occupations have three common characteristics of emotional labor: (a) public face-to-face or voice-to-voice contact; (b) production of an emotional state in another person; and (c) a degree of control of emotional activities by the employer. In relation to the current study, the workers are subjected to the demands of emotional labor when they connect with survivors face-to-face or voice-to-voice to listen, discuss, and address their challenges with IPV. While workers have to process the horrific stories of emotional and physical abuse to women on a daily basis—leaving workers depressed, disheartened, or emotionally drained—they must suppress or manage these emotions while performing their role so survivors can feel a sense of safety, being cared for, and trusted. Unfortunately, the findings indicate that this is rarely recognized or acknowledged by employers as a means of producing stress on the job that carries into workers’ personal lives. Groggel's (2023) study explored this concept among American IPV legal service workers and reported comparable findings in that workers carried the emotional burden of processing survivor's traumatic narratives, and tried to hide their distress during support to manage their workload. Similarly, Tracy's (2005) research that focused on the perspectives of American correctional officers suggested that in order for one to be effective in their role, workers need to construct an emotional facade, which may result in psychological discomfort when workers’ “emotional performances conflict with real feelings” (p. 273). In congruence with the female participants in the current study, previous studies highlight how women tend to hold occupations that involve care work (Wharton, 2009) and jobs that involve increased emotional labor are commonly thought to be “women's work” (Harris, 2024, p. 68). Both studies identified comparable consequences among emotional laborers including burnout, low job satisfaction, increased job stress, and feelings of depersonalization (Harris, 2024; Wharton, 2009). Additionally, how CWs in this study perceive their emotional labor to be devalued is also problematic and as Harris (2024) suggests, it is important for employers to acknowledge and appreciate the existence of emotional labor. Although employee turnover rates were not directly measured in the current study, all workers—including novice and senior workers—indicated that their organizations are grappling to hire workers because nonprofit sectors offer better pay and a work–life balance. However, nonprofits are limited in terms of the process of securing ongoing funding and relying on government grants to operate. In fact, American and Canadian gender-based violence organizations have historically been underfunded and vulnerable to budget cuts (Alani & Stroink, 2015; Sapire et al., 2022). As CWs in this study and what previous literature suggests, a reason for this may reflect a systemic gender equality issue because these organizations are predominantly run by women to support women (Fernandes & Lanthier, 2024; Rossiter et al., 2020). Extant literature also suggests that gender-based systemic inequalities, such as how societies often devalue, minimize, or deny women who experience violence, may erode the well-being of workers who serve this population as they feel their work is perceived as devalued (Fernandes & Lanthier, 2024). Considering these risks, it may be of value for community IPV organizations to collaborate with local public/community health units, hospital domestic violence departments, or mental health associations as a means to promote community awareness about the health consequences of CWs’ emotional labor and initiate primary prevention strategies that will support the needs of the IPV CW workforce. Likewise, a gap in knowledge and skills related to complex mental health and addiction cases was identified by CWs. Implementing a Train of Trainer (TT) model, where mental health and addiction experts may train CWs, could be useful in building internal capacity to navigate these complex cases. The TT model has the potential for greater outreach without burden of cost in facilitating mental health training (Frank et al., 2020).

While it is not known if CWs’ work–life boundaries were influential, it is interesting to note that nearly half of the CWs reported that they were separated or divorced from their intimate partners. This finding reinforces how gender may play a role in contributing to CWs’ emotional turbulence as scholarship suggests that female IPV workers self-identified with abused women, which led to their own intimate partner conflicts (Bell, 2003; Goldblatt et al., 2009). This study highlighted how the emotional work of supporting survivors can deplete CWs’ emotional and mental capacity for loved ones, and how CWs struggled to separate survivors’ experiences from their way of seeing the world. In these cases, implementing strategies that support vicarious posttraumatic growth, such as continuous social support and employee debriefing after difficult interactions, can provide a means to help manage emotional responses and grow from work-related secondary trauma (Deaton et al., 2021). The findings in this study support Ellis and Knight's (2018) research on empathetic engagement and their model of secondary trauma among victim service providers (including sexual and domestic violence advocates, law enforcement officials, healthcare providers, and child protective service workers). Ellis and Knight (2018) describe how empathetic engagement may be used as a tactic by IPV service workers to better understand survivors’ situations and therefore be able to effectively support them. However, similar to the present study, this engagement may influence workers’ inability to set effective boundaries between their work and personal lives, disrupt their ability to control emotional responses, and affect their sense of trust with others (Ellis & Knight, 2018). Research demonstrates that support with maintaining professional boundaries could also be a strategy to help CWs separate work from their personal life (Deaton et al., 2021; Wagaman et al., 2015). This may include limiting self-disclosure with survivors, scheduling frequent breaks between cases, engaging in grounding activities, or like what some CWs reported in this study, reducing indirect trauma exposure during personal time by limiting or avoiding violent movies, podcasts, and television.

Unfortunately, CWs in this study similarly struggled with feeling helpless, demoralized, and lost motivation in their work; this may be related to structural factors not within their control. Considering the complexity of issues faced by CWs, there may be opportunities to capitalize on resources in the local context by considering the primary health care (PHC) principles and strengthening intersectoral collaboration (World Health Organization, 2024). Health professionals in various sectors—domestic violence, community, mental, or public health—may work toward strengthening partnerships with IPV organizations and coalitions to collectively develop strategies to support CWs in their role. To date, the health and wellness of CWs have been somewhat invisible. Guided by the PHC principles, promoting health and well-being may be achieved by: (a) creating awareness of the health consequences of CWs’ work experiences; (b) reinforcing the importance of CWs’ services in assisting survivors; (c) collaborating to facilitate mental health and addictions education and training for CWs; and (d) advocating and lobbying for improvements in shelter/housing options for IPV survivors across all levels of government.

The findings are of significance to IPV organizations, healthcare professionals, and others working in the IPV field to inform on the health and wellness outcomes and recommendations for the improvement of CWs’ work-life. Addressing the needs of CWs is timely because of the exponential rise in the number of IPV cases against women since the COVID-19 pandemic (Women and Gender Equality Canada, n.d.). Further research is needed to examine CWs’ roles, experiences, and organizational structure in a larger Canadian context; this knowledge will provide a greater scope of issues faced by CWs in local contexts. It may be valuable for future researchers to measure employee turnover rates and if there are differences in voice versus senior employees leaving their position in nonprofit IPV organizations. Furthermore, it will advance our understanding and inform the development of tailored support strategies for CWs, that promote health, and in turn, this may encourage worker retention in the IPV field.

### Strengths and Limitations

The strength of this study includes the diversity of CWs’ roles across four unique organizations servicing IPV survivors. While the study was conducted in a region in Ontario, Canada, findings may be transferrable to similar contexts and populations in other settings. While the study demonstrated a high degree of credibility as CWs had diverse supports, workplace practices, and coping strategies, most of the sample consisted of White/Caucasian female CWs. The findings may have been different if a more diverse sample was obtained. Further, background data on the survivors that CWs served was not collected as the study's focus was on CWs’ experiences. However, this information may be of value for future researchers to consider to better understand how CWs who may have personal experiences with similar trauma cope in their role.

## Conclusion

This study contributes knowledge to the field of IPV as the findings demonstrated several ways in which supporting IPV survivors influenced the health and wellness of Canadian CWs in nonprofits. The findings shed light on how CWs’ work felt unmanageable, created emotional distress, harmful physical symptoms, and social disruptions. Additionally, there is advanced understanding of how CWs manage stressful work experiences, which highlighted several strategies that would assist in promoting better health and wellness outcomes for CWs.
